# F45. THE EFFICACY AND SAFETY OF BLONASERIN AFTER SWITCHING FROM OTHER ATYPICAL ANTIPSYCHOTICS IN SCHIZOPHRENIC INPATIENTS: AN OPEN-LABEL, MULTI-CENTER TRIAL

**DOI:** 10.1093/schbul/sby017.576

**Published:** 2018-04-01

**Authors:** Bo-Hyun Yoon, Won-Myong Bahk, Young Joon Kwon, Sang-Yeol Lee, Kwanghun Lee, Moon Doo Kim, Sung-Yong Park, Min-Kyu Song

**Affiliations:** 1 Naju National Hospital; 2 St. Mary’s Hospital, The Catholic University of Korea; 3 College of Medicine, Soonchunhyang University; 4 Wonkwang University, School of Medicine; 5 College of Medicine, Dongguk University; 6 School of Medicine, Jeju National University; 7 Keyo Hospital; 8 Yesan Mental Health Clinic

## Abstract

**Background:**

The aim of this study was to investigate the efficacy and safety of blonanserin treatment after switching from other atypical antipsychotics in schizophrenic inpatients who showed inadequate efficacy and poor tolerability.

**Methods:**

A total of 63 schizophrenic inpatients (inadequate response group=45 and poor tolerability group=18) were included in this study. They were already treated with atypical antipsychotics except blonanserin and not favored due to inadequate responses or intolerable adverse effects. Blonanserin was administered during 12 weeks after switching from their previous antispsychotics. Treatment response was evaluated with Brief Psychiatric Rating Scale (BPRS) and CGI-S, and safety profile were measured with Abnormal Involuntary Movement Scale (AIMS), Simpson-Angus Extrapyramidal Side effects Scale (SAR)S and Barnes Akathisia Rating Scale (BARS). Drug Attitude Inventory (DAI-10) and Subjective Well-being Under Neuroleptic Treatment (SWN) were used for subjective estimates. Assessments were done at baseline, 1, 2, 4, 8 and 12 weeks after blonanserin treatment. Repeated measures of ANOVA were done to analyze the group (inadequate vs. intolerable group) and time effects.

**Results:**

CGI and BPRS were showed significant treatment responses after switching to Blonaserin. Time effects were significant at 2, 4, 8, 12 weeks after switching and group by time effect were also significant at that time. Mean changes of AIMS, SARS and BARS scores were not significant throughout test trial. Although SWN was significantly improved after switching to Blonaserin, it was not found significant group by time effect.

**Discussion:**

The results suggest that blonanserin may be effective and well tolerable in schizophrenic patients who showed inadequate treatment response or poor tolerability.

